# Association of a variable number tandem repeat in the *NLRP3* gene in women with susceptibility to RVVC

**DOI:** 10.1007/s10096-016-2600-5

**Published:** 2016-03-07

**Authors:** M. Jaeger, A. Carvalho, C. Cunha, T. S. Plantinga, F. van de Veerdonk, M. Puccetti, C. Galosi, L. A. B. Joosten, B. Dupont, B. J. Kullberg, J. D. Sobel, L. Romani, M. G. Netea

**Affiliations:** Department of Internal Medicine and Radboudumc Center for Infectious Diseases, Radboud University Medical Center, P.O. Box 9101, 6500 HB Nijmegen, The Netherlands; Department of Experimental Medicine, University of Perugia, Polo Unico Sant’Andrea delle Fratte, 06132 Perugia, Italy; Hôpital Necker, Paris, France; Department of Medicine, Infectious Diseases, Wayne State University School of Medicine, Detroit, MI USA

## Abstract

Vaginal infections with *Candida* spp. frequently occur in women of childbearing age. A small proportion of these women experience recurrent vulvovaginal candidosis (RVVC), which is characterized by at least three episodes of infection in one year. In addition to known risk factors such as antibiotics, diabetes, or pregnancy, host genetic variation and inflammatory pathways such as the IL-1/Th17 axis have been reported to play a substantial role in the pathogenesis of RVVC. In this study, we assessed a variable number tandem repeat (VNTR) polymorphism in the *NLRP3* gene that encodes a component of the inflammasome, processing the proinflammatory cytokines IL-1β and IL-18. A total of 270 RVVC patients and 583 healthy controls were analyzed, and increased diseases susceptibility was associated with the presence of the 12/9 genotype. Furthermore, functional studies demonstrate that IL-1β production at the vaginal surface is higher in RVVC patients bearing the 12/9 genotype compared to controls, whereas IL-1Ra levels were decreased and IL-18 levels remained unchanged. These findings suggest that IL-1β-mediated hyperinflammation conveyed by the *NLRP3* gene plays a causal role in the pathogenesis of RVVC and may identify this pathway as a potential therapeutic target in the disease.

## Introduction

Vulvovaginal candidosis is one of the most frequent infections in women. *Candida* spp. in general and *C. albicans* in particular are the main cause of this infection. Although not life-threatening, vulvovaginal candidosis leads to severe discomfort, manifested by itching, soreness, rash, and vaginal discharge [1]. Antifungal medication leads to improvement of the health status in these women, but a small portion of women continue to experience recurrent vaginal infections with the fungus, a condition described as recurrent vulvovaginal candidosis (RVVC). Up to 6–8 % of all women are known to suffer from RVVC, defined by at least three periods of vaginal *Candida* infection per year [2–4]. Although glucocorticosteroid use, immunosuppressive and antibiotic therapy, or pregnancy are known risk factors for RVVC, most patients lack any of these risk factors.

This led to extensive research to identify the (multi-)genic components predisposing to RVVC susceptibility. Several variants in genes known to be important in the innate host defense have been found to be associated with RVVC susceptibility [5–10]. A whole range of genetic variations in the *NLRP3* gene have previously been shown to be associated with inflammatory diseases [11–13]. *NLRP3* is part of a multicomplex cytoplasmic protein platform called inflammasome, which allows the activation of caspase-1. In turn, caspase-1 cleaves the inactive precursors of interleukin-1 beta (IL-1β) and IL-18 into the biologically active cytokines. A variable number tandem repeat (VNTR) in intron 4 (rs74163773) of the *NLRP3* gene has previously been shown to be associated with essential hypertension, as well as (self-reported history of) vaginal *C. albicans* infections, and a reduction of IL-1β levels in patients suffering from vulvar vestibulitis syndrome [14, 15]. The present study aims to assess the role of the VNTR in *NLRP3* in patients suffering from RVVC, as well as to investigate the functional consequences of this sequence variant on the specific host defense against *C. albicans* at the vaginal surface.

## Materials and methods

Patients were recruited at the Santa Maria della Misericordia Medical Center (Perugia, Italy), Wayne State University School of Medicine (Detroit, MI, USA), Radboud University Medical Center (Nijmegen, The Netherlands), and Hôpital Necker (Paris, France). Patients were enrolled during episodes of acute vaginitis or, if asymptomatic, while receiving maintenance fluconazole therapy. Inclusion criteria for the study were: otherwise healthy women of at least 18 years of age, diagnosed with at least three microbiologically documented episodes of vulvovaginal candidosis within one year, all caused by *C. albicans*. Exclusion criteria were use of any immunosuppressive therapy (including steroids), diabetes, pregnancy, or human immunodeficiency virus (HIV) infection. EDTA venous blood was collected after obtaining written informed consent. The study was approved by the Institutional Review Boards of each of the study centers, and enrollment took place between January 2009 until December 2013. All patients and controls were of self-reported Western European genetic background. To confirm the genetic homogeneity between samples, we performed multidimensional scaling (MDS) analysis based on 550,000 single nucleotide polymorphisms (SNPs) and excluded outliers.

### Genotyping

Genomic DNA was isolated from ETDA venous blood using the Gentra Puregene Blood Kit following the manufacturer’s standard protocol (Qiagen, Venlo, The Netherlands). To amplify the 42-bp VNTR in intron 4 (rs74163773) of the *NLRP3* gene, we used conventional polymerase chain reaction (PCR) with a primer pair already described by Omi et al. [16]. The resulting amplicons were subsequently loaded on a 3 % agarose gel to perform electrophoresis. In our setup, we found five different alleles, namely 6, 7, 9, 12, and 13, forming the following eight genotypes: 12/13, 12/12, 12/9, 12/7, 12/6, 9/9, 7/9, and 7/7. The 12 allele was reported to consist of 720 bp, while the 9, 7, and 6 alleles consisted of 594, 510, and 468 bp, respectively [16]. In addition to this, we observed an additional allele just 42 bp above the 720-bp 12 allele, named allele 13 (762 bp), in line with Omi et al. [15]. Differences between patients and controls in each of the two cohorts were determined using the χ^2^ statistical analysis.

### Cytokine measurement in vaginal fluids

Cervicovaginal samples were collected as described earlier by Snowhite et al. [17]. In brief, samples were obtained from all participants during episodes of vaginosis by instilling 3 ml of sterile saline into the posterior vagina, mixing the saline with secretions and withdrawing the solution with a 10-ml plastic syringe. No needles or flushes were used. All vaginal washes were centrifuged at 12,000 × g for 10 min to separate the mucus from the PBS wash solution shortly after collection, and pellet fractions were immediately frozen at −80 °C. Cytokine levels in the lavage fluids were determined by ELISA (R&D Systems). Data were normalized to total protein levels for each sample using the Bio-Rad Protein Assay (Bio-Rad, Milan, Italy) and expressed as log_10_ pg cytokine/μg total protein).

## Results

**Fig. 1:**
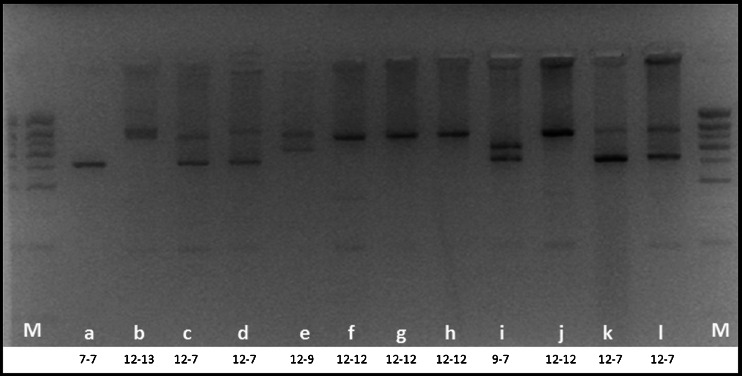
Example of different genotypes of the *NLRP3* variable number tandem repeat (VNTR) polymorphism on a 3 % agarose gel. Allele 13: 762 bp, allele 12: 720 bp, allele 9: 594 bp, allele 7: 510 bp, and allele 6: 468 bp; M = 100-bp marker

In our experimental setup, the 42-bp VNTR within intron 4 (rs74163773) of the *NLRP3* gene has eight genotypes derived from five different alleles. The genotypes appear in different bands on an agarose gel, as shown in Fig. 1. The 12/12 genotype was the most prevalent in both control and patient groups, while the 12/7, 12/9, and 7/7 genotypes were less prevalent. The 12/13, 12/6, 7/9, and 9/9 genotypes were less abundant, together accounting for around 15 % of the genotypes present. Except for the 12/9 genotype, we observed minor differences comparing the different genotypes between controls and patients. Nevertheless, significant differences were seen when we compared the 12/9 genotype between patients and controls (*p*-value 0.02) (Table 1).

**Table 1:** Genotype distribution of the *NLRP3* genotypes for the variable number tandem repeat (VNTR) in intron 4

*NLRP3* genotype	Controls		RVVC patients		*p*-Value
	*n*	%	*n*	%	
12/12	308	52.83	134	49.63	0.43
12/13	9	1.54	7	2.59	0.44
12/7	134	22.98	62	22.96	1.00
12/9	52	8.92	39	14.44	0.02
12/6	3	0.51	2	0.74	1.00
7/7	45	7.72	11	4.07	0.06
7/9	22	3.77	11	4.07	0.98
9/9	10	1.72	4	1.48	1.00
Total	583		270		

**Fig. 2:**
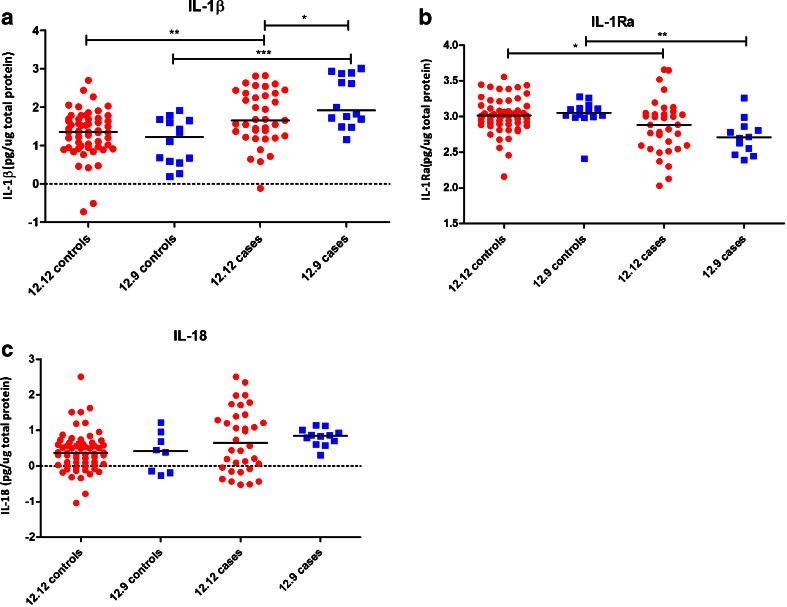
Log_10_ cytokines (IL-1β, IL-1Ra, and IL-18 in pg cytokine/μg total protein) in vaginal fluid from recurrent vulvovaginal candidosis (RVVC) patients and controls. **a** IL-1β levels between healthy controls and RVVC patients bearing either the 12/12 (*red*) or the 12/9 (*blue*) genotype. **b** IL-1Ra levels. **c** IL-18 levels. Data were analyzed using the Mann–Whitney U test and * indicates significance of *p* ≤ 0.05, **p ≤ 0.01, and ***p ≤ 0.001

To investigate the local immune response at the mucosal surface, we measured inflammasome-dependent cytokine concentrations in vaginal fluids from either controls or patients suffering from RVVC. In the analysis, we concentrated on the 12/12 wild-type genotype and the 12/9 genotype that was found to be associated with RVVC. IL-1β levels were higher in the vaginal fluid of RVVC patients compared to healthy controls. In addition, the 12/9 genotype led to even higher IL-1β concentrations compared to the 12/12 genotype. On the other hand, IL-1Ra levels were lower in patients for both genotypes compared to controls, while those with the 12/9 genotype had lower concentrations than the patients with the 12/12 genotype. Differences in IL-18 levels did not reveal significant differences between patients and controls, or between the RVVC patients with different genotypes (Fig. 2).

## Comments

A small study has reported a specific genotype distribution of a VNTR in the *NLRP3* gene in patients with vaginal candidosis [14, 16]. If confirmed, this finding would have important consequences for our understanding of RVVC, as it would identify cytokines of the IL-1 family as a potential therapeutic target in the disease. Moreover, a crucial aspect that needed to be elucidated was whether the risk allele was associated with a higher or lower cytokine production capacity at the level of vaginal mucosa. In the present study, we report that the 12/9 genotype of the rs74163773 VNTR in *NLRP3* was associated with recurrent vulvovaginal infections with *C. albicans*. Moreover, we report differential production of the inflammasome-associated cytokines between patients and controls, with the patients bearing the risk allele displaying the highest concentration of IL-1β in the vaginal fluid.

The *NLRP3* polymorphism rs74163773 results in 11 genotypes derived from five different alleles. In our setup, we found only eight of the 11 genotypes, which might be the result of the low prevalence of the other three genotypes within the Caucasian population [15]. A previous study found allele 7 of the *NLRP3* gene to be associated with an increased frequency of RVVC [14]. We were not able to validate this association; however, we observed that the 12/9 genotype is significantly associated with an increased susceptibility to RVVC. Both studies argue thus that genetic variation in *NLRP3* may influence the occurrence of vulvovaginal candidosis, but larger future studies are needed in order to validate this and discern the exact genotypes involved.

In the vaginal fluid, IL-1β cytokine concentrations were higher in RVVC patients compared to controls. Furthermore, carriage of the 12/9 genotype led to an even higher IL-1β concentration in the vaginal fluid. In line with this, we found an inverse correlation between IL-1β and IL-1Ra concentrations, with IL-1Ra levels being lower in patient samples and even further decreased in patients carrying the 12/9 genotype. An increase in IL-1β levels accompanied by a decreased IL-1Ra concentration in patients bearing the 12/9 genotype may lead to a hyperinflammatory state at the vaginal surface [18]. This is in line with the concept that RVVC is likely due to an inappropriate immunological reaction to *Candida* growth and colonization, rather than an immune defect resulting in fungal overgrowth. There are several important recent findings that strengthen this conclusion, and propose that the inflammasome/IL-1β pathway as an important mediator for the inflammatory reaction:

i. The yeast-to-hyphae germination has been recently shown to be an important component of vulvovaginal candidosis pathogenesis [19], and earlier studies demonstrated that inflammasome/IL-1β activation is a main component of the hyphae-specific immune response [20].
ii. A recent transcriptome study of murine vaginal candidosis has identified the *NLRP3* inflammasome of a strong feature of the *Candida*-induced transcription profile in the vagina [21].
iii. The vaginal fluid showed continuous increases in neutrophils and IL-1β of vaginally infected mice during the late phases of infection, despite a stable fungal load [19].

In the present study, we present strong evidence that a similar overactivation of the inflammasome and IL-1β (but not IL-18) production is present in patients with RVVC, and genetic variation in the *NLRP3* inflammasome gene has an important impact on this mechanism.

In conclusion, this study reveals new insights into genetic susceptibility factors that contribute to the development of RVVC. Defects in the inflammasome component *NLRP3* are involved in disease development and progression, most likely by influencing *Candida*-induced cytokines towards a hyperinflammatory state. It is important to emphasize that RVVC is a multifactorial disease, in which not only genetics and the innate immune system, but also the local microbiome, metabolome as well as external factors likely play a substantial role. However, understanding genetic and local defects may increase our knowledge of the exact pathological mechanisms and contributes to the development of a better diagnosis and novel therapies. In this respect, agents targeting IL-1β overproduction may prove useful in the management of RVVC.
